# Aggressive sellar-suprasellar atypical teratoid rhabdoid tumor in a 38-year-old pregnant woman: a case report and literature review

**DOI:** 10.1097/MS9.0000000000003382

**Published:** 2025-05-26

**Authors:** Habeeb H. Awwad, Anas K. Assi, Zaina A. Khaled, Riman A. Sultan, Sara N. Fatoum, Mohammad Hakam Shehadeh, Haneen Owienah, Mohand Abulilhya, Sali Salman, Nadeem Shain, Abdalwahab Kharousha, Hadi Dababseh

**Affiliations:** a Faculty of Medicine, Al-Quds University, Jerusalem, Palestine; b Department of radiology, Al-istishari Arab Hospital, Ramallah, West Bank, Palestine; c Pathology Department, Al-istishari Arab Hospital, Ramallah, West Bank, Palestine; d Department of Neurosurgery, Al-istishari Arab Hospital, Ramallah, West Bank, Palestine

**Keywords:** atypical teratoid rhabdoid tumor (ATRT), INI1 (SMARCB1) loss, pregnancy, recurrence, tumor resection

## Abstract

**Introduction and importance:**

Atypical teratoid rhabdoid tumor (ATRT) is a rare and aggressive central nervous system malignancy. It primarily affects infants and young children, with adult cases being exceptionally uncommon. This case highlights the diagnostic and management challenges posed by ATRT in a 38-year-old pregnant woman.

**Case presentation:**

We present a case of ATRT in a 38-year-old pregnant woman, highlighting the diagnostic and management challenges posed by this aggressive tumor during pregnancy. A sellar-suprasellar lesion during the third trimester of pregnancy was discovered. Following neurosurgical evaluation and imaging studies, a diagnosis of ATRT was confirmed by histopathological examination. The patient underwent a left fronto-lateral craniotomy for tumor resection. Afterward a viable baby was delivered to proceed with adjuvant management.

**Clinical discussion:**

ATRT is an aggressive tumor mainly affecting infants, but with rare adult cases linked to inactivation of SMARCB1 (INI1) or SMARCA4 (BRG1). ATRT is characterized by a very poor prognosis. A multimodal treatment approach, combining surgery, chemotherapy, and radiotherapy has been associated with better survival rates. As unusual, maximal safe resection was achieved in our patient, but adjuvant therapy was deferred due to pregnancy, and highlighting the difficult balance between maternal and fetal health in such cases.

**Conclusion:**

This case highlights the aggressive nature of ATRT in adults, the challenges of managing the tumor during pregnancy, and the critical need for early recognition and intervention to optimize outcomes. Further research is essential to improve prognosis in ATRT cases, particularly in unique clinical scenarios like pregnancy.

## Introduction

Atypical teratoid/rhabdoid tumor (ATRT) is a rare and highly aggressive central nervous system (CNS) malignancy. It predominantly affects infants and young children, with the majority of cases occurring in those under three years of age^[1]^. It is exceedingly rare in adults, with cases in individuals over 18 years old are uncommon, with a lifetime risk estimated to be less than 1 in 1 000 000^[2]^. The clinical presentation of ATRT in adults is highly variable and often nonspecific, depending on the tumor’s location within the CNS. Symptoms may include headaches, nausea, vomiting, focal neurological deficits, or signs of increased intracranial pressure^[3]^. Diagnosing ATRT in adults poses significant challenges due to the rarity and overlapping radiological and histological features with other CNS malignancies, such as glioblastoma, medulloblastoma, or metastatic tumors,^[4]^. Accurate diagnosis relies on a combination of histopathological examination, immunohistochemical staining for INI1 or BRG1 loss, and molecular genetic testing^[1,3]^.

**HIGHLIGHTS**

- Though primarily a pediatric tumor, atypical teratoid rhabdoid tumor should be considered in adult patients with aggressive central nervous system tumors.
- Symptoms like headaches and vomiting can be misattributed to pregnancy, delaying tumor diagnosis.
- Treatment decisions must balance maternal tumor progression and fetal viability, often delaying adjuvant therapy.

The management of adult ATRT remains poorly defined, largely due to the scarcity of reported cases and the lack of standardized treatment protocols. Current therapeutic approaches are extrapolated from pediatric protocols and typically involve maximal safe surgical resection, followed by adjuvant radiotherapy and chemotherapy^[5]^. However, the prognosis for adult ATRT patients remains poor, with a median overall survival ranging from 6 to 18 months^[6]^, highlighting the need for more effective treatment strategies and a deeper understanding of the tumor’s biology in this age group.

In this report, we present a case of ATRT in a 38-year-old pregnant woman, illustrating the clinical challenges in diagnosing and managing this aggressive tumor during pregnancy. This case report has been reported in line with the SCARE 2023 criteria^[7]^.

## Case presentation

A 38-year-old female, G3P2A0, at 31 weeks of gestation, was referred from a local hospital for neurosurgical evaluation because of a sellar-suprasellar lesion. Her medical history was unremarkable, except for two previous cesarean sections. She had no prior history of neurological or systemic illnesses. The patient reported progressive headaches over the past month that were initially tolerable and responsive to paracetamol. Two weeks prior to admission, her headache worsened significantly and was accompanied by recurrent vomiting that was unresponsive to antiemetics. On 17 November 2024, she woke up with diplopia and left eye ptosis. Concerned about her worsening symptoms and weight loss, she sought medical care at a local hospital. She was admitted there for intravenous (IV) antibiotics, analgesia, IV fluids, and antiemetics. Despite these interventions, her symptoms persisted. A brain MRI performed on 20 November 2024 that revealed a sellar-suprasellar lesion with features suggestive of an aggressive tumor. She was referred to our hospital after two days for further evaluation and management.

Upon admission on 22 November 2024, the patient was alert and oriented with a Glasgow Coma Scale (GCS) score of 15/15. Neurological examination revealed dilated and nonreactive left pupil, cranial nerve findings revealed left eye ptosis with diplopia when using both eyes. Extraocular muscle movement was intact bilaterally. Visual acuity was normal in both eyes with counting fingers from 2 meters; slight blurring in the left eye. Motor examination revealed normal power in all limbs with no pronation or drift, and Romberg’s test was positive. A detailed endocrinological evaluation revealed a non-functioning pituitary adenoma-like hormonal profile. And gynecological consultation confirmed a viable fetus at 31 + 4 weeks gestation with an estimated fetal weight of 1800 grams.

**Figure 1.**
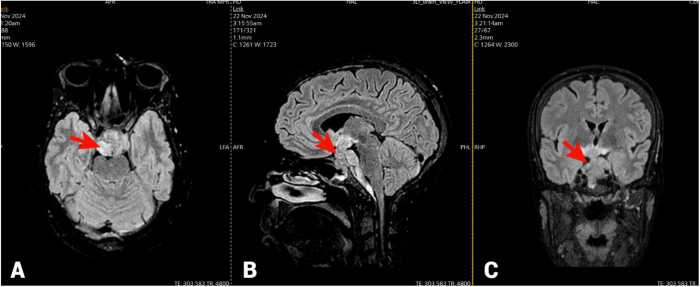
Brain MRI without IV contrast of the brain demonstrating: (A) axial, (B) sagittal, and (C) coronal views. There is a heterogeneous sellar and suprasellar mass with high signal intensity on T2W/FLAIR sequences measuring about (3.3 × 2.4 × 4 cm) and posterior extension measures 6 mm in thickness (red arrows). MRI: magnetic resonance imaging; IV: intravenous.

A brain MRI using time of flight angiography sequences without IV contrast showed a heterogeneous sellar and suprasellar mass with low signal intensity on T1W and high signal intensity on T2W/FLAIR sequences. SWI revealed multiple foci suggesting micro-hemorrhages or possible calcifications (Fig. 1). The mass measured about 3.3 × 2.4 × 4 cm with posterior extension measuring 6 mm in thickness. It encircled the right internal carotid artery (ICA) without narrowing it and contacted the medial side of left ICA. It likely obliterated both cavernous sinuses, while other dural venous sinuses remained open without thrombosis.

The mass showed invasive characteristics, extending posteroinferiorly to the foramina magnum with a mass effect on the pons and basilar artery without invasion. Superiorly, with upward displacement of the lamina terminalis involves the floor of the third ventricle and interpeduncular cistern, and inferiorly, it involves the sphenoid sinus. High T2/FLAIR and low T1 signals along the optic tracts in the thalamic region suggested edema with significant mass effect to the optic chiasm with splaying of the optic tracts.

On 24 November 2024, a left fronto-lateral craniotomy was scheduled for microsurgical resection of the lesion under neuromonitoring. The surgical goal was maximal safe resection while preserving critical structures. Gross total resection was not achievable due to tumor infiltration along the clivus and cavernous sinus. The patient underwent general anesthesia with continuous cardiotocography for fetal heart rate to ensure fetal well-being.

The Surgery was performed without complications. After that, the patient was extubated and transferred to the ICU for close observation and management. Neurologically she recovered full consciousness with a GCS score of 15/15, normal speech, intact memory, normal power at all limbs, intact sensation and reflexes. A visual exam of both eyes resolved left oculomotor partial palsy (improved ptosis, pupil reactive). There was also intact extraocular muscle movement and visual acuity in both eyes with slight blurring of vision in left eye, and right mild homonymous hemianopia in the right eye. Central diabetes insipidus developed postoperatively, and was effectively managed with desmopressin (Minirin) and hydrocortisone stress dosing.

**Figure 2.**
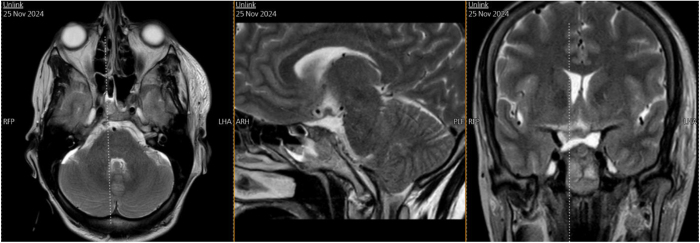
Brain MRI without IV contrast of the brain demonstrating: (A) axial, (B) sagittal, and (C) coronal views. Partial resection of the previously described sellar mass is seen.

Postoperatively brain MRI without IV contrast was done and showed postoperative changes at the site of surgery in the sellar region in the form of edematous changes, multiple foci of blooming artifacts suggesting hemorrhagic spots and pneumocephalus. Right frontotemporal scalp edema was also noted. Bilateral abnormal high (fluid attenuated inversion recovery) FLAIR rim (mostly subdural hematoma) in the posterior fossa. Partial resection of the previously described sellar mass is seen (Fig. 2).

**Figure 3.**
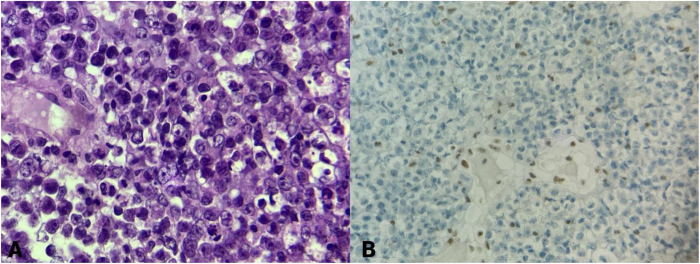
Sellar-suprasellar lesion biopsy. (A) This slide shows a microscopic description of the tumor, which is composed of undifferentiated, small, and pleomorphic cells arranged in sheets and clusters. The cells exhibit hyperchromatic nuclei, prominent nucleoli, and a high mitotic rate. Many of the tumor cells demonstrate an irregular shape and show a rhabdoid phenotype, characterized by eccentrically placed nuclei and large nucleoli. These cells are interspersed with areas of necrosis and hemorrhage. (B) This immunohistochemistry slide reveals negative (loss of expression) INI1 (SMARCB1).

Pathology evaluation of the tumor was performed and microscopic description revealed the tumor composed of undifferentiated, small, and pleomorphic cells arranged in sheets and clusters. The cells exhibited hyperchromatic nuclei, prominent nucleoli, and a high mitotic rate. Many tumor cells demonstrated an irregular shape and showed a rhabdoid phenotype, characterized by eccentrically placed nuclei and large nucleoli. These cells were interspersed with areas of necrosis and hemorrhage (Fig. 3A).

Immunohistochemistry revealed negative (loss of expression) INI1 (SMARCB1), Synaptophysin was scattered focal positivity, focal expression of Keratin in some areas, and scattered focal positivity of glial fibrillary acidic protein. The loss of INI1 (SMARCB1) protein expression was consistent with an ATRT, confirming the diagnosis (Fig. 3B).

This loss of INI1 (SMARCB1) expression was a key finding in the diagnosis of ATRT, which is an aggressive and highly malignant embryonal tumor. Further genetic testing was recommended for a broader understanding of the patient’s molecular profile, including the presence of SMARCB1 mutations or other genetic syndromes that predispose patients to rhabdoid tumors.

On 1 December 2024, a cesarean section was performed under spinal anesthesia to ensure maternal safety while preparing for adjuvant radiotherapy. The surgery went well with born of a viable baby. And the patient recovered well post-delivery with stable vital signs and no major complications. She remained under close observation until December 4, when she was discharged in good condition with plans for outpatient follow-up.

On 16 December 2024 a CT scan was done and showed a huge recurrence of Tumor. So, the patient was admitted to our ward still having persistent headache and looking ill. Her neurological exam showed dilated, non-reactive left pupil (consensual response present only in right) with ptosis, and counting fingers in right from 2 Meters. She had temporal hemianopsia on the right, ophthalmoplegia on the left, and she couldn’t raise her eyebrow on the left. Also, she had a positive Romberg test.

**Figure 4.**
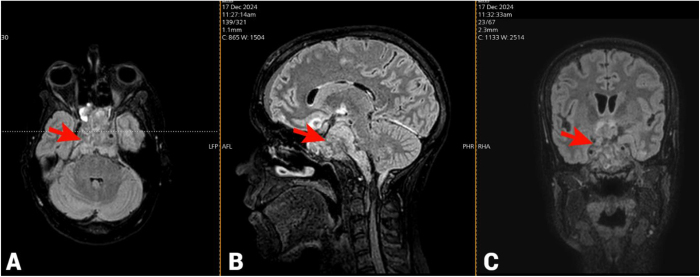
Brain MRI with T2W/FLAIR sequences of the brain demonstrating evidence of disease recurrence: (A) axial, (B) sagittal, and (C) coronal views. There is a large heterogeneous sellar and suprasellar mass with heterogeneously high signal intensity on T2W/FLAIR sequences. The mass appears invasive and extending in different directions. The sellar-suprasellar bulk measuring about 4.2 × 4.5 × 4.5 cm, posterior extension measures 1.5 cm in thickness (red arrows). MRI: magnetic resonance imaging.

The patient underwent brain MRI which showed recurrent hemorrhagic part of the lesion with significant compression and infiltration of both cavernous sinuses. So, the team planned a Left Frontal Craniotomy for microsurgical maximal safe resection of malignant Seller and Suprasellar lesion for the next day (Fig. 4).

Intraoperatively, the tumor was found extensively infiltrating all basal cisterns, invading the dura, and forming dense adhesions between the dura and brain cortex. It occupied the entire subarachnoid space, encased the ICA and optic nerve, and obliterated anatomical landmarks, making advancement through the subarachnoid spaces and cisterns impossible. The mass was presented as a block, preventing brain retraction without significant risk of injury to cranial nerves or major blood vessels at the skull base.

Given the high risk of fatal complications associated with surgical resection and the poor prognosis of the condition, our surgical team held an intraoperative discussion with the patient’s immediate family (mother and brother). They were informed about the severe risks of proceeding with the surgery. After understanding the situation, the family opted to discontinue the procedure to avoid the high likelihood of intraoperative or postoperative mortality. A palliative approach was mutually agreed upon.

Three days postoperatively the patient developed a sudden onset decrease in the level of consciousness, she also had bilateral dilated pupils nonreactive to light and no response to pain or stimulus. There was no corneal, gag or cough reflex. Apnea test was positive and her GCS was 3/15. She was diagnosed with brain death with very poor and critical condition, and she died on the next day.

## Discussion

ATRT is an aggressive tumor primarily affecting infants, with rare adult cases linked to inactivation of SMARCB1 (INI1) or SMARCA4 (BRG1)^[8]^. Recent studies indicate a shift in adult ATRT locations, with the sellar region now more common (41.9–46.9%) than the cerebral hemispheres (21.9–35.14%)^[8]^. Sellar ATRT exclusively affects females (94.7%) with a median age of 40, while ATRTs in other regions typically affect younger individuals and show male predominance^[5,9]^. Although data on ATRT in adults is generally limited, studies involving pregnant women with ATRT are even scarcer with only a few cases of ATRT during pregnancy have been reported. Studies suggest that hormonal changes during pregnancy may be the main contributor to the aggressive behavior of the tumor and may accelerate tumor proliferation in hormone-sensitive neoplasms. This highlights the need for further research on pregnant women, to address the diagnostic and therapeutic challenges associated with such cases. Also, this case contributes to the growing incidence of ATRT in the sellar region, aiming to achieve improved outcomes in managing this aggressive tumor in comparable cases.

ATRTs’ clinical features are nonspecific and depend on their location and mass effects on the CNS. Common symptoms include vomiting and nausea due to increased intracranial pressure^[10]^. Sellar or suprasellar ATRT may present with headache and visual disturbances, mimicking pituitary macroadenomas, especially when compressing the optic nerve^[8]^. A key distinguishing feature is the rapid symptom progression, headaches and vision changes developing over weeks to months, contrasting with slower progression in pituitary macroadenomas^[5]^. Our patient’s rapid symptom progression, including diplopia and ptosis, underscored the tumor’s aggressiveness. Cerebellar symptoms or cranial nerve palsies (VI and VII) may occur if the tumor involves the cerebellum and cerebellar-pontine angle^[10]^.

Diagnosing ATRT in adults is challenging due to overlapping imaging and clinical features with high-grade glioma, pineoblastoma, germinoma, pituitary macroadenoma, and other embryonal tumors^[4]^. Common imaging findings include hemorrhage, calcifications and cavernous sinus or clivus invasion in sellar cases^[10]^. Gadolinium-enhanced MRI, the gold standard, is essential for diagnosis and should include the entire neuraxis to detect leptomeningeal disease, present in up to 35% of cases^[10,11]^. On CT, ATRTs appear as heterogeneous hyper-dense masses with peripheral calcifications^[10]^; on MRI, they are large, heterogeneous tumors, hypodense on T1, hyperdense on T2, and often show restricted diffusion on DW1^[11]^. ATRTs frequently invade dura and bone, unlike most intramedullary brain tumors, disregarding natural anatomical planes^[10]^, and histopathology is typically required for definitive diagnosis.

Histologically, ATRTs show cells with clear cytoplasm, prominent nucleoli, and rhabdoid cells, often with a staghorn vasculature pattern^[5,8]^. Immunohistochemically, loss of INI1 nuclear staining is diagnostic, supported by markers like vimentin and EMA^[12]^. The potential association between pregnancy and ATRT may involve indirect mechanisms, such as steroid hormone interaction with INI1 and growth factor-induced cytokeratin 8 phosphorylation, rather than direct hormonal effects^[12]^. This association is relevant in our pregnant patient and may have contributed to the tumor’s rapid growth and recurrence, though further research is needed.

Genetically, chromosome 22 abnormalities cause SMARCB1/INI1 loss in 70% of cases, with rare SMARCA4 involvement^[11,13]^. Our patient’s INI1 loss suggests a SMARCB1 mutation. SMARCB1 C-terminal mutations are linked with better outcomes, while SMARCA4 mutations are more aggressive^[13]^. Sellar ATRTs often match the ATRT-MYC methylation profile, aiding personalized therapy^[5]^. DNA methylation divides ATRTs into three subgroups, with ATRT-TYR showing better survival rate in children^[11]^. High Ki-67% (>35%) predicts poor outcomes^[8]^. Diagnosis requires genetic testing, immunohistochemistry, and methylation profile, but limited access to these tools highlights the need for further research^[11]^. This limitation was evident in our case, where advanced molecular testing was not performed.

Overall, due to its rapid growth and spread in cerebrospinal fluid, ATRT is characterized by a very poor prognosis^[14,15]^. In adults, the mean survival rate ranges from 0 to 120 months^[8]^. As reported, sellar ATRT appears to have slightly better prognosis and overall survival compared to pediatric ATRT and adult ATRT occurring in other locations^[8]^. Although maximal safe resection was attempted, our patient experienced a massive tumor recurrence within two weeks, emphasizing the aggressive nature of ATRT. Comparatively, in other adult ATRT cases, recurrence rates vary, but such rapid progression post-resection is uncommon.

A multimodal treatment approach, combining maximal safe surgery, chemotherapy, and radiotherapy, has been associated with better survival rates, with early initiation of therapy being crucial for optimizing prognosis^[5]^. In our patient, maximal safe resection was achieved, but adjuvant therapy was deferred due to pregnancy, likely contributing to the tumor’s aggressive recurrence, highlighting the difficult balance between maternal and fetal health in such cases. However, the absence of standardized therapeutic protocols for adults in clinical practice remains a significant challenge. Individuals who underwent adjuvant chemotherapy and radiation therapy had obviously longer survival durations than those who did not, despite the fact that adjuvant treatment types varied widely^[8]^.

The most common surgical approach for these tumors is the transsphenoidal approach^[8]^. Adjuvant treatment regimens for adult sellar ATRT varied widely across reported cases, high-lighting the lack of a standardized chemotherapy protocol for this condition^[5]^. Commonly used chemotherapy regimens include combinations of agents such as vincristine, doxorubicin, cyclophosphamide or ifosfamide, carboplatin, and etoposide^[8]^.

Compared to previously published cases, our case stands out due to the convergence of multiple high-risk factors including pregnancy, sellar-suprasellar location, rapid recurrence, and limited therapeutic options. It emphasizes the critical need for early diagnosis, multidisciplinary collaboration, and standardized treatment protocols for adult ATRT, particularly in the context of pregnancy.

## Conclusion

In conclusion, this case highlights the complexities and challenges in diagnosing and managing ATRT in adults, particularly in the context of pregnancy, to optimize outcomes for both mother and fetus. The rapid progression of symptoms and the aggressive nature of the tumor highlight the critical need for early recognition and intervention. Despite achieving maximal safe resection, the patient’s poor prognosis reiterates the importance of developing standardized treatment protocols for adult ATRT. Future research should focus on enhancing diagnostic accuracy, understanding the molecular underpinnings of this malignancy, and exploring effective therapeutic strategies to improve outcomes for affected individuals.

## Data Availability

None.

## References

[1] Karim A Shaikhyzada K Suleimenova A. Case report: atypical teratoid/rhabdoid tumor of the lateral ventricle in a male adolescent (case-based review and diagnostic challenges in developing countries). Front Oncol 2022;12:985862. 36276064 10.3389/fonc.2022.985862 PMC9582653

[2] Woehrer A Slavc I Waldhoer T. Incidence of atypical teratoid/rhabdoid tumors in children. Cancer 2010;116:5725–32. 20737418 10.1002/cncr.25540

[3] Ismail S Ghanem L Ibrahim L. Atypical teratoid/rhabdoid tumor of the central nervous system: clinicopathological features of two challenging cases. Int J Surg Case Rep 2024;117:109531. 38507938 10.1016/j.ijscr.2024.109531 PMC10963598

[4] Korshunov A Sturm D Ryzhova M. Embryonal tumor with abundant neuropil and true rosettes (ETANTR), ependymoblastoma, and medulloepithelioma share molecular similarity and comprise a single clinicopathological entity. Acta Neuropathol 2014;128:279–89. 24337497 10.1007/s00401-013-1228-0 PMC4102829

[5] Major K Daggubati LC Mau C. Sellar atypical teratoid/rhabdoid tumors (AT/RT): a systematic review and case illustration. Cureus 2022;14:e26838. 35974867 10.7759/cureus.26838 PMC9375109

[6] Buscariollo DL Park HS Roberts KB. Survival outcomes in atypical teratoid rhabdoid tumor for patients undergoing radiotherapy in a Surveillance, Epidemiology, and End Results analysis. Cancer 2012;118:4212–19. 22213196 10.1002/cncr.27373

[7] Sohrabi C Mathew G Maria N. The SCARE 2023 guideline: updating consensus Surgical CAse REport (SCARE) guidelines. Int J Surg Lond Engl 2023;109:1136. 10.1097/JS9.0000000000000373 PMC10389401 37013953

[8] Georgountzos G Gkalonakis I Kyriakopoulos G. A rare case of atypical teratoid rhabdoid tumor at the sellar region in an adult: case report and review of literature. Brain Spine 2024;4:104138. 39634169 10.1016/j.bas.2024.104138 PMC11616529

[9] Corvino S De Caro MD Franca RA. Atypical teratoid/rhabdoid tumor of the nervous system in adults: location-related features and outcome. World Neurosurg 2023;179:e404–e415. 37659753 10.1016/j.wneu.2023.08.107

[10] Calandrelli R Massimi L Pilato F. Atypical teratoid rhabdoid tumor: proposal of a diagnostic pathway based on clinical features and neuroimaging findings. Diagnostics 2023;13:475. 36766580 10.3390/diagnostics13030475 PMC9914341

[11] Nesvick CL Nageswara Rao AA Raghunathan A. Case-based review: atypical teratoid/rhabdoid tumor. Neurooncol Pract 2019;6:163–78. 31386032 10.1093/nop/npy037 PMC6656328

[12] Erickson ML Johnson R Bannykh SI. Malignant rhabdoid tumor in a pregnant adult female: literature review of central nervous system rhabdoid tumors. J Neurooncol 2005;74:311–19. 16132523 10.1007/s11060-004-7560-4

[13] Chan V Marro A Findlay JM. A systematic review of atypical teratoid rhabdoid tumor in adults. Front Oncol 2018;8:567. 30547013 10.3389/fonc.2018.00567 PMC6279935

[14] Paun L Lavé A Jannelli G. Pediatric posterior fossa ATRT: a case report, new treatment strategies and perspectives. Brain Sci 2023;13:712. 37239184 10.3390/brainsci13050712 PMC10216074

[15] Peng AJ Fan SC Chen YX. Atypical teratoid/rhabdoid tumor in adult: case series and an integrated survival analysis. Br J Neurosurg 2021;38:425–32. 33595416 10.1080/02688697.2021.1885620

